# Profiling of Current Transients in Capacitor Type Diamond Sensors

**DOI:** 10.3390/s150613424

**Published:** 2015-06-08

**Authors:** Eugenijus Gaubas, Tomas Ceponis, Dovile Meskauskaite, Nikolai Kazuchits

**Affiliations:** 1 Institute of Applied Research, Vilnius University, Sauletekio av. 9-III, LT-10222 Vilnius, Lithuania; E-Mails: tomas.ceponis@ff.vu.lt (T.C.); dovile.meskauskaite@ff.stud.vu.lt (D.M.); 2 Belarusian State University, Nezavisimosti av. 4, 220030 Minsk, Belarus; E-Mail: kazuchits@bsu.by

**Keywords:** HPHT and CVD grown diamond, carrier lifetime, drift mobility, ambipolar diffusion

## Abstract

The operational characteristics of capacitor-type detectors based on HPHT and CVD diamond have been investigated using perpendicular and parallel injection of carrier domain regimes. Simulations of the drift-diffusion current transients have been implemented by using dynamic models based on Shockley-Ramo’s theorem, under injection of localized surface domains and of bulk charge carriers. The bipolar drift-diffusion regimes have been analyzed for the photo-induced bulk domain (packet) of excess carriers. The surface charge formation and polarization effects dependent on detector biasing voltage have been revealed. The screening effects ascribed to surface charge and to dynamics of extraction of the injected bulk excess carrier domain have been separated and explained. The parameters of drift mobility of the electrons μ*_e_* = 4000 cm^2^/Vs and holes μ*_h_* = 3800 cm^2^/Vs have been evaluated for CVD diamond using the perpendicular profiling of currents. The coefficient of carrier ambipolar diffusion *D_a_* = 97 cm^2^/s and the carrier recombination lifetime τ*_R,CVD_* ≌ 110 ns in CVD diamond were extracted by combining analysis of the transients of the sensor current and the microwave probed photoconductivity. The carrier trapping with inherent lifetime τ*_R,HPHT_* ≌ 2 ns prevails in HPHT diamond.

## 1. Introduction

Diamond is one of the most promising wide-gap materials for applications in the fabrication of high frequency sensors and radiation-tolerant particle detectors [1,2,3,4,5,6,7,8,9,10]. The excess carrier lifetime and carrier mobility as well as diffusion coefficient are ones of the most sensitive parameters to electrically active defects in materials. The carrier lifetime is the main limiting factor for charge collection efficiency in diamond-based sensors. Manifestation of these parameters is most pronounced in the operational dynamics of devices fabricated using synthetic diamonds made by different technologies [11,12,13,14,15]. The examined operation of capacitor type detectors [13,14,15,16,17] confirmed the possibility of fabricating fast and rather radiation-resistant sensors relative to traditional devices produced on silicon [18,19,20,21,22,23,24]. The operation dynamics and charge transport characteristics are traditionally examined by measurements of the transients of the injected charge drift current (ICDC) [13,15,18] and analysis of these characteristics [16,17,18,25,26] based on Shockley-Ramo’s theorem [27,28]. These methods based on pulsed current characterization are often generalized as the transient current (TCT) [15] or time-of-flight (TOF) [18] techniques. These techniques are most suitable for evaluation of the performance of device structures fabricated by different technologies and for defect characterization as well as of their influence on the transport properties of the excess charge carriers in diamond. Despite significant progress in synthetic diamond growth and device fabrication technologies, the material characteristics and operation dynamics of the devices fabricated using synthetic diamonds manufactured using different technology (HPHT, CVD) still need to be unveiled and understood. The items to study in this context include polarization effects [16,29], the role of surface termination in its charging and surface conductivity [30,31,32], possibilities of surface passivation [33] and regimes for relevant formation of electrodes [34].

In this work, a few aspects of performance of the capacitor-type detectors made of different technology diamonds have been examined. Profiling of the microwave-probed photoconductivity transients has been performed for evaluation of the lateral distribution of recombination centres and for extraction of carrier recombination lifetimes. The prevailing recombination processes with carrier lifetimes in the range of τ*_R,HPHT_* ≌ 2 ns and significant (>40%) lateral dispersion of these values were revealed in HPHT diamond structures, while considerably longer carrier recombination lifetimes of τ*_R,CVD_* ≌ 110 ns with nearly invariable values over wafer plane were obtained in CVD diamond structures. CVD and HPHT diamond samples, that exhibit considerably different carrier lifetimes, were chosen to analyze detector signals attributed to carrier recombination and drift current components. The dynamics of the polarization effect, which is commonly ascribed to ionized traps [16], have been revealed and their dependence on bias voltage as well as on bulk and localized excess carrier injection regime has been analyzed. This polarization effect appeared to be significant in the range of rather low (<50 V) bias voltages by affecting the electric field in sensors operating with high repetition rate. Profiling of the detector current pulses originated from bulk injection of excess carriers within the inter-electrode gap has been performed by varying the bias voltage. Ultraviolet (354 nm) light laser pulses of sub-nanosecond duration were employed to produce rather depth-homogeneous excess carrier injection within the laser beam. Profiling of the detector currents has also been performed by varying the initial position of the injected local domains of excess carriers within the inter-electrode space of detector, where the directions of the applied electric field and of a sharply focused laser beam are perpendicular. The parallel and perpendicular geometry of orientations of electric field and excitation beam were thereby combined for versatile examination of the current transients. This enabled us to separate the regimes of prevailing carrier drift, ambipolar diffusion and recombination processes and to evaluate their parameters. The parameters of drift mobility have been evaluated μ*_e_* = 4000 cm^2^/Vs for electrons and μ*_h_* = 3800 cm^2^/Vs for holes, respectively, in CVD diamond using profiling of currents under perpendicular geometry regime. The averaged carrier mobility of μ = 2700 cm^2^/Vs in CVD diamond has also been estimated from voltage profiling of drift current transients using a flat vertex approximation for the measured current pulsed transients. The coefficient of carrier ambipolar diffusion *D_a_* = 97 cm^2^/s in CVD diamond was extracted by combining an analysis of the transients of the sensor current and the microwave-probed photoconductivity. The depth-distribution of the grown-in defects in HPHT diamond wafers has also been identified within the profiles of current transients. The recombination current component prevailed for detectors made of the examined HPHT diamond samples.

## 2. Samples and Experimental Regimes 

To get recordable detector signals with the typical parameters of carrier pair generation (of <100 pair/μm) within wide band-gap materials, a rather thick active width of detectors is needed to register high energy (penetrative) particles by collecting the radiation injected charge. On the other hand, formation of junctions in wide band-gap materials, such as diamond, GaN, *etc*., addresses technological problems concerning selection and insertion of the relevant dopants with rather shallow levels. Leakage currents of particle sensors should be small, as well. Therefore, capacitor type detectors made of diamond are widely used [1,2,3,4,5,6,7,8,9,10,11,12,13,14,15,16,17,18]. 

In this work, the single-pixel capacitor-type detectors were formed by using high pressure high temperature (HPHT) and chemical vapour deposition (CVD) grown diamond materials and different metallization types in the fabrication of electrodes.

The carrier lifetime (τ*_R_*) evaluations, using the microwave-probed photoconductivity (MW-PC) technique [35], were initially performed on HPHT and CVD diamond samples. Values of the τ*_R_* of about 2 ns and of 110 ns were obtained for HPHT and CVD diamond samples, respectively. A rather homogeneous carrier lifetime lateral distribution has been obtained for a CVD Element Six sample under bulk (354 nm UV) excess carrier excitation using 400 ps laser pulses, while inhomogeneous lateral distribution of carrier lifetime (with about 40% dispersion of values) was deduced from microwave-probed photoconductivity (MW-PC) scans in HPHT diamond wafers, which correlated with lateral maps of the electrically active impurity distribution [35]. 

**Figure 1:**
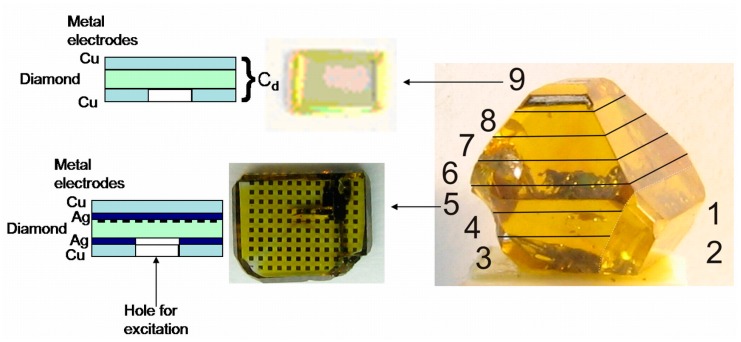
A sketch of slicing of the HPHT diamond crystal and a picture of the micro-pixel (isle type) contacts deposited on the wafer sample No. 5. The cross-sections of the capacitor type sensors made of diamond wafers covered by metallic electrodes are also shown in the figure.

The HPHT diamond single crystals, synthesized by the high pressure (4.5–5.0 GPa) and high (1350–1450 °С) temperature gradient technology [36] using a Ni-Fe-C liquid solvent/catalyst carbon metallurgy system, were investigated. The growth regimes and preparation of the samples numbered from 1 to 9 are described in more detail in [35]. Octahedral growth planes prevailed on the as-grown crystal, while cube growth sectors were clearly observed on the crystal-top plane situated opposite to a seed. Wafers No. 1 and No. 2 [35] were cut in parallel to this line (Figure 1). The samples from No. 3 to No. 9 (Figure 1) were prepared by slicing the residual part of the crystal into wafers across the plane (001) parallel to the crystal base. The 250–500 μm thick two-side mechanically polished wafers have surface roughnesses of less than 20 nm. For wafer No. 4 [35], complicated projections of different morphology sectors grown from a seed and metallic inclusions were also observed. The octahedral sectors dominate in wafers No. 4 and No. 7 [35], while the internal cube growth morphology sectors of small areas were resolved. The superposition/intersection of several cube and octahedral sectors were revealed within a central area of wafer No. 4 [35] located above a seed. Pressed copper electrodes, compatible with micro-strip line formed on printed circuit board (PCB), or two-component silver (Ag) and copper (Cu) metallization were employed to fabricate parallel-plate capacitor-type sensor structures (Figure 1). These structures with different type electrodes were examined in order to clarify the impact of diamond surface states and the role of carrier traps in appearance of the dynamic polarization effect.

A mosaic electrode system has additionally been made on the sample No. 5 (Figure 1) of the HPHT wafer set, which contains a rather large concentration of metallic inclusions. This wafer sample was thicker, d = 748 μm. Isle (micro-pixel)-type electrodes of dimensions of 200 × 200 μm^2^ were produced by implantation of boron (B) proceeded by annealing under vacuum. These 200 μm step contact isles covering the entire surface of wafer sample were employed for the characterization of micro-pixel electrodes. The linear current-voltage dependence for each single micro-pixel was obtained for applied voltages of up to a few hundred volts over all the set of micro-pixels within the wafer planar surface, while dark current values varied in the range from a few fA to a pA. These variations have been ascribed to the lateral distribution of impurities spread within the bulk of this wafer [35], where current variations correlated well with carrier lifetime lateral maps on the wafer surface. These B implanted contacts showed rather good stability and were resistant to thermal and chemical treatments. Then, pressed copper electrodes or two-layer metal electrodes (Figure 1) were applied to form a parallel-plate capacitor type sensor on this (No. 5) HPHT diamond sample.

**Figure 2:**
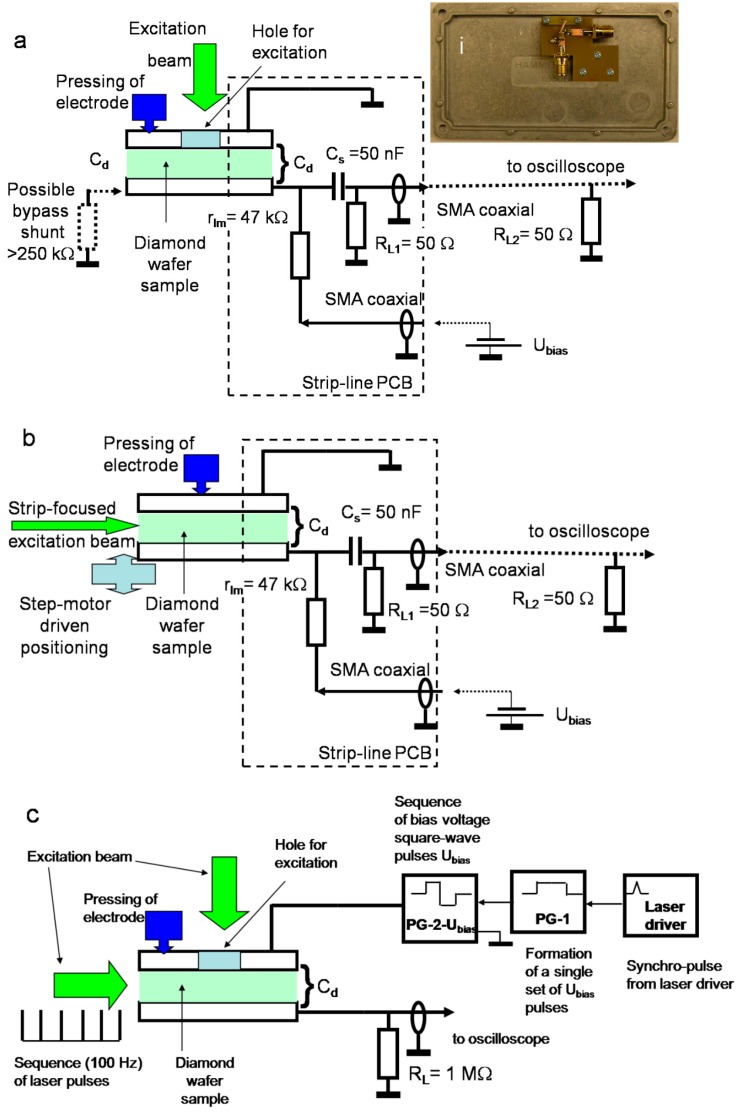
Schemes exploited for the recording of current transients using the parallel (**a**) and the perpendicular (**b**) profiling regimes are represented in Figure 2a,b, respectively. The DC bias voltage, varying bypass shunt has been applied within the circuit (**a**) for the investigation of relaxation features of the polarization effect. A picture of the PCB is also illustrated in the inset for Figure 2a. A scheme for examination of the origin and relaxation characteristics of the polarization effect by using pulsed applied voltage synchronized with a sequence of laser excitation pulses is shown in (**c**). Here PG denotes a pulse voltage generator.

Capacitor type structures on HPHT wafer samples No. 4, No. 7 and No. 9 (Figure 1) using pressed plate electrodes (Figure 2) were made for comparison. These capacitor type structures were also formed in such a geometry so as to be compatible with a strip-line printed circuit board (PCB) dimensions (inset of Figure 2a), exploited in measurements of the injected charge current transients.

Single-crystal wafer samples of CVD diamond (Element Six commercial material [37]) of dimensions 3 × 3 mm^2^ and 300 and 500 μm thick, were also involved into measurements. These (100) oriented single-crystal CVD diamond wafers possessed impurities of nitrogen (N) and boron (B) of concentrations <5 ppb and <1 ppb, respectively. The pressed plate copper electrodes (Figure 1 and Figure 2) used in the formation of capacitor type CVD diamond structures were exploited to maintain the same experimental conditions as those employed for investigations of HPHT diamond structures. 

The capacitor type structures were mounted on a strip-line PCB with equivalent *R_L_* = 25 Ω load resistances. The strip-line PCB (pictured within the inset of Figure 2a) is inevitable for the reliable measurements of short current pulses in materials with large carrier mobility and in order to reduce electrical noises. A conventional electrical scheme for the TCT measurements (e.g., [13,14,15,16,38,39]) was employed, and measurements were performed at room temperature. Here, the *r_lm_* = 47 kΩ resistance is used for limitation of the *C_S_* = 50 nF capacitor charging current. For biasing of the diamond filled capacitor *C_d_* under test device (CUD), CUD is connected in series with a direct current (DC) voltage *U* source and *r_lm_.* A circuit for pulsed signal is then comprised of the charged source capacitor *C_S_*, of the sample capacitor *C_d_* and the load resistor *R_L1_* = 50 Ω (on input of coaxial cable) connected in series. These elements *C_d_*, *C_S_*, and *R_L1_* are mounted (within shielded compartment) on the strip-line PCB to have the matched and short wire connections. The 50 Ω coaxial cable is loaded by *R_L2_* = 50 Ω on its output and at input of a TDS-5104 1 GHz band digital oscilloscope, to get equivalent *R_L_* = *R_L1_||R_L2_* = 25 Ω. A closed input of the oscilloscope is discriminated by 12 pF capacitor from the DC signals. The time parameters, evaluated for the electrical and excitation pulse rise, lead then to the overall time-resolution *Δt* ≌ 500 ps, in our experiments.

UV light (354 nm) laser pulses of 400 ps were mainly used for injection of the excess carrier domains (packets) in the CVD and HPHT diamond samples. Optical 400 ps pulses of 531 nm were also exploited for excitation when carrier generation is possible through impurity levels in HPHT diamond. Two measurement geometries have been employed: a parallel one (Figure 2a), when the excitation beam and the applied electric field directions are parallel and a perpendicular one (Figure 2b), when the excitation beam impinges perpendicularly to the applied field direction; there the beam direction is nearly parallel to the *C_d_* capacitor plates. The injected charge drift current (ICDC) transients are profiled by the applied DC voltage for the parallel measurement geometry. 

The bulk domain of excess carriers is injected in diamond for parallel measurement geometry by using pulsed laser light quanta of *h*ω *< E_G_* = 5.6 eV. The excitation is then performed through the transparent electrode (or a hole within a metal electrode of about 1 mm diameter) in parallel measurement geometry. The lateral diffusion within excess carrier packet (initially generated through electrode hole) can be ignored if transit time is significantly shorter than diffusion time.

The inherent carrier diffusion time considerably exceeds the transit time when the lateral wafer dimensions are large in comparison with wafer thickness. The non-focused laser beam (Figure 2a,c) can also be used for the bias voltage-based profiling of currents by injection of bulk excess carrier domain if a diameter of the perpendicularly impinging beam either covers or is close to a dimension of the inter-electrode gap. On the other hand, the localized (surface plane) charge domain is injected by a sharply focused laser beam for the perpendicular measurement geometry. A strip-shape beam sharply focused by a cylindrical lens is exploited in the latter case, to avoid the transverse gradients in the injected excess carrier domain. 

To clarify the origin of a polarization effect, modifications of the measurement circuits were employed either by using a shunt of rather large resistance (Figure 2a) or pulsed biasing voltage (Figure 2c). The pulse bias voltage, as a sequence of either unipolar or bipolar square-wave pulses, of various durations that significantly exceed a laser pulse repetition rate (100 Hz) was applied (Figure 2c), in order to monitor CUD charging currents and relaxation characteristics of their amplitudes due to polarization effects. To govern synchronization, delays and formation of a rather long chain of square-wave pulses, triggered by a single laser pulse, two pulse generators (PG, Figure 2c) were exploited. The first one (PG-1) synchronized from a single laser pulse has been used to form a long (from a few of ms to several seconds) pulse, which governs the generator (PG-2) of square-wave pulses of voltage in the range of 10–16 V. The latter PG-2 generates a chain (from two to several waves of the desirable duration) of square-waves of either unipolar or bipolar voltage pulses. Duration and quantity of pulses in the chain is gated by the parameters of a pulse of the first generator (PG-1). The CUD charging currents, as a chain of pulses of varied amplitude attributed to a sequence of the identical laser light pulses of 100 Hz rate, are recorded by an oscilloscope using 1 MΩ load resistance.

Measurements of the pulsed optical transmission as a function of excitation power density and dependence of electrical response signals on excitation intensity were performed to reveal the dominant carrier generation processes. One ns temporal resolution DET-10A Si detectors and calibrated optical PM-100D power-meter were used in the transmission measurements. The geometrical parameters of the focused laser beams were evaluated by using knife-edge and CCD camera-based beam-shape profilers. The single side outspread at a 0.1*I_0_* level of light intensity within focused beam is about of 20 μm relative to the peak intensity *I_0_*. A homogeneous domain (packet) of excess carriers (in a plane parallel to the capacitor electrodes), localized within a waist of the focused laser beam, is injected. In the case of the perpendicular measurement geometry, profiling of the drift current transients is performed by changing the position of the injected domain within the inter-electrode space. It can easily be deduced that the role of diffusion within formation of current transients is different for the parallel and perpendicular profiling geometries. Therefore, the models of description of the injected carrier drift current (ICDC) transients should initially be considered to understand and interpret the obtained profiling results.

## 3. Models for Analysis of Drift-Diffusion Dynamics

Consideration, based on Shockley-Ramo’s theorem and applied to the ICDC transients under injection of the localized domains, is presented more detail in our publications [38,39,40]. Actually, the electron-hole neutral domain is initially injected in most cases of carrier excitation either as the secondary carrier pairs generated by high energy particles or photo-excited carrier pairs. The electric field acting on drifting electron and hole sub-domains, depends on the instantaneous position of the drifting sub-domains. This field is determined by solving the Poisson’s equation including the charge on electrodes, induced by an external voltage source, and the charge induced by the moving charge sub-domains. The current transient detected on a load resistance within external loop of circuit is then varied in time due to charge changes on electrode caused by the injected domain drift, where the external source should balance variations of charge on electrode according to [28]. Instantaneous current values are then determined by the charge variations in time (integrated over an area of electrode) related to the characteristic transit time.

**Figure 3:**
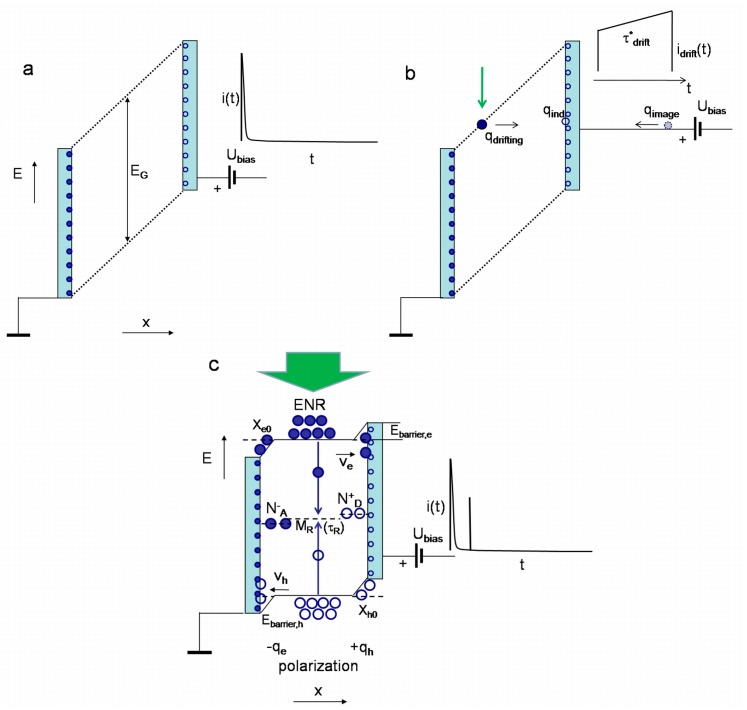
Sketches of the changes of the induced charges and of energy band diagram (*E*) within a capacitor structure filled with diamond (of band-gap *E_G_*). (**a**) Charges (small circles: hollow -positive, solid- negative charges) induced on electrodes just after switching-on of the DC external voltage. On the right, a current transient of charging of the capacitor sensor is sketched; (**b**) Sketch of the monopolar drift phase for the light injected charge (large circle) and formation of drift current pulse (on the right). Here, the induced charge on electrode and external current (image charge) during drift are also sketched; (**c**) Sketch of the polarization charge (−*q_e,_ q_h_*) formation under both the excess carrier trapping onto deep centres (quasi-stationary polarization) and formation of the (*X_e0_, X_h0_*) depletion (from one type of excess carriers) regions at electrodes (dynamic polarization) after drifting (*v_e_, v_h_*) excess carriers arrive to electrodes. In the electrically neutral region (ENR), recombination (τ*_R_*) through centres *M_R_* is possible, while charged centres (*N^−^_A_, N^+^_D_*) can appear due to carrier trapping. On the right, a current transient of charging (of a photo-capacitance effect) of the capacitor sensor is sketched.

**Figure 4:**
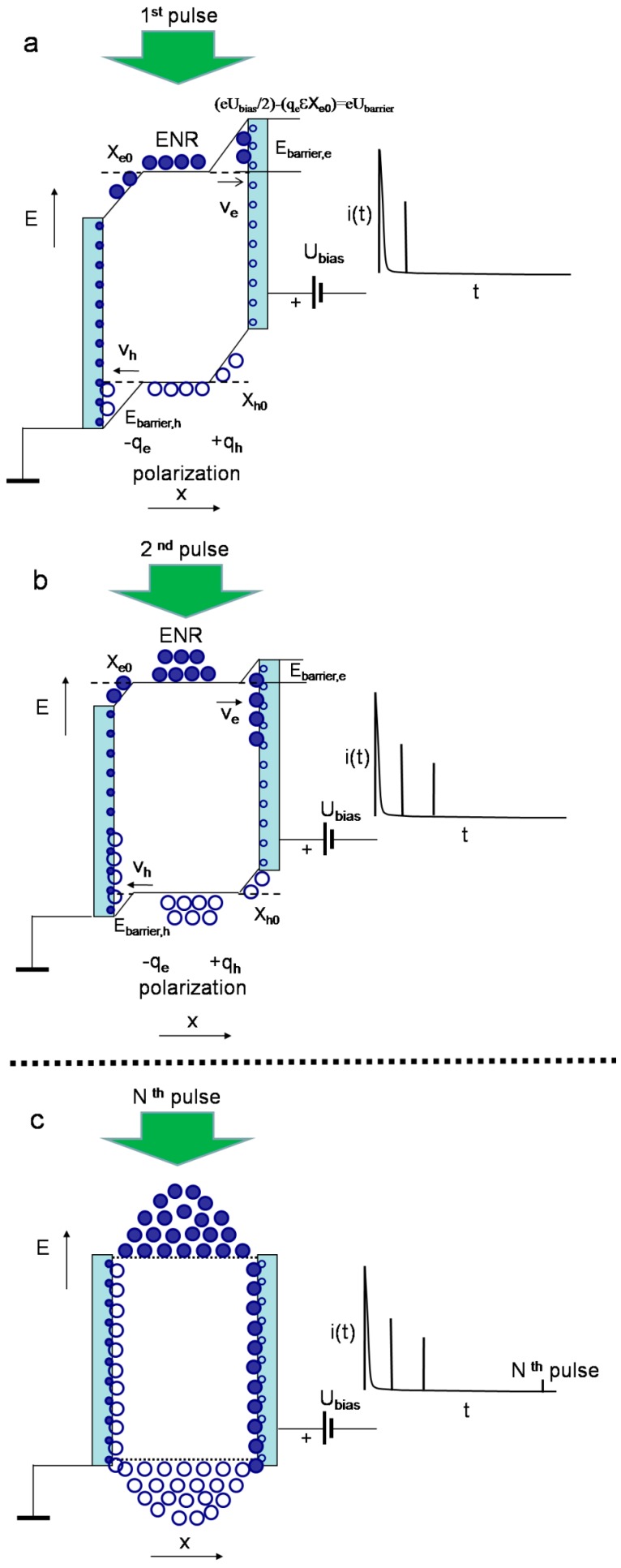
Sketches of evolution of the light injected charges (large circles) under switched-on external voltage of rather small value and of the dynamic polarization through accumulation of the space charge under a set of light pulses (1st—**a**, 2nd—**b**, and *N*-th—**c**) those inject bulk density of excess carrier pairs (hollow and solid large circles). The dynamic depletion widths are denoted by *X_e0_* and *X_h0_*. The diagrams of the capacitor charging currents (vertical lines) after each *N*-th light pulse are there shown on the right.

**Figure 5:**
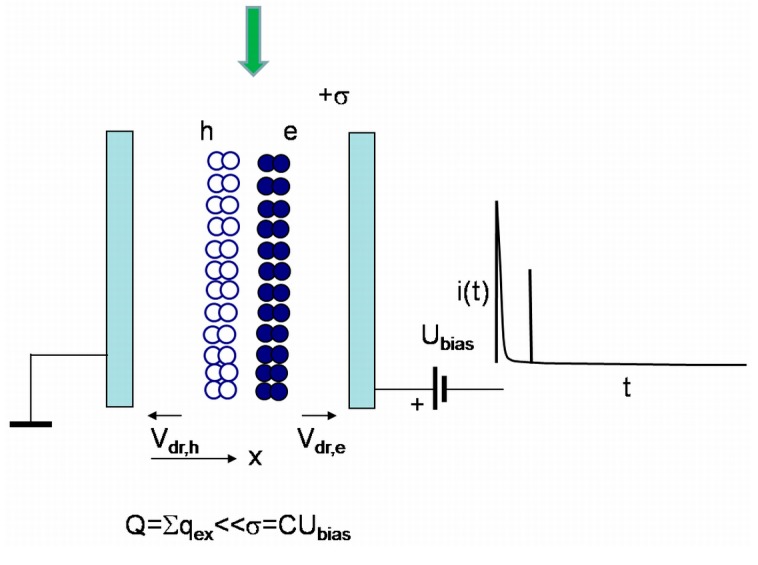
Sketch of separation and drift of the light locally injected charges (of rather small amount) under switched-on external voltage of value capable to separate excess carrier pairs. Here, energy band diagram is skipped. This sketch illustrates the drift prevailing processes at moderate and high applied voltages. The main notifications and assumptions are the same as for Figure 3. On the right, a diagram of capacitor charging and of drift current pulses is sketched.

**Figure 6:**
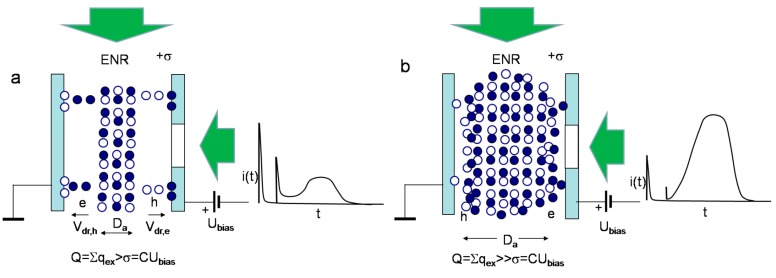
Sketches of diffusion current formation under large amount of the light injected carrier pairs, when a partial or complete screening of the external field by injected carriers appears. (**a**) Formation of the partial dynamic depletion layers, separated by ENR-electrically neutral region (filled with non-separated excess carriers) which supplies diffusing carriers from ENR (for drift within near-electrode regions), during formation of a current pulse under the light injected bulk charge. Energy diagram is here skipped. On the right, a diagram of capacitor charging, of drift current and of diffusion component pulses is sketched; (**b**) Sketch of the diffusion prevailing current pulse under bulk injection of the large amount of the excess carrier pairs which charge is capable to completely screen the external field. Notifications and assumptions are the same as for Figure 3.

Simplified sketches of the processes and the formation regimes for current pulses are illustrated in Figure 3, Figure 4, Figure 5 and Figure 6. In Figure 3, the energy and charge distributions are sketched for sensor biasing (a), for the phase of monopolar drift of the injected excess carriers (b), and for the polarization charge formation (c), under both the excess carrier trapping to deep centres (quasi-stationary polarization) and formation of the depletion (from one type of excess carriers) region at electrodes (dynamic polarization) after drifting excess carriers arrive to electrodes. Sketches of the evolution of the light injected bulk charge distribution and of the dynamic polarization through accumulation of the space charge under a set of light pulses those inject bulk density of excess carrier pairs are illustrated in Figure 4. In Figure 5, a sketch of separation and drift of the locally injected charges (of rather small amount) under switched-on external voltage of value capable to separate excess carrier pairs is presented. In Figure 6, sketches of the diffusion current formation under large amount of the light injected carrier pairs in the bulk of material within inter-electrode gap are illustrated, when a partial or complete screening of the external field by injected carriers appears.

The transit time for the drift prevailing processes is evaluated by solving the equation for the instantaneous drift velocity normalized to the whole drift region length. It had been shown [38,39,40] that there are a vast variety of regimes (Figure 3, Figure 4, Figure 5 and Figure 6) such as pure bipolar and mixed (bipolar-proceeded by the monopolar) drift processes and drift proceeded by diffusion. The role of diffusion can be ignored if an injected charge does not exceed the charge on electrode induced by the external voltage source (Figure 4 and Figure 5). On the contrary, the injected charge surface domain is able to screen the electric field caused by a fixed value of the bias voltage if the external voltage is low and the surface density of the injected charge is large (Figure 6). This regime is ascribed to the diffusion limited convection current—the current transient appears as a result of the time varied charge, diffused to and collected on electrode.

### 3.1. Models for Injection of a Bulk Domain at Low Applied Voltages

The current transients can be caused by the time varied bulk excess charge domain, overwhelming the inter-electrode space, which disappears due to diffusion to and collection on electrode (Figure 4 and Figure 6). This happens if applied external field is insufficient (due to low voltage) to separate the electron-hole sub-domains over the entire bulk. The latter case is the most complicated for consideration. Then, the carrier reservoir (diffusion there determines a fraction of the charge carriers driven by electric field) is located within the inter-electrode gap, and the diffusion supplied carriers replace the extracted to electrode ones.

The charge of non-extracted carrier surface sub-domain (due to inappropriate polarity of external field) screens the external field (Figure 4), and, thus, determines a characteristic length of charged layer. The same charged layer appears at the opposite electrode. These opposite charged layers determine an additional electric field, equivalent to Dember field for ambipolar diffusion. This Dember-like field also prevents diffusion of the non-extracted carriers to electrodes at the opposite sides (Figure 4). The analytical description of current transients, induced by a bulk electron-hole domain which cannot be disassembled owing to the applied external electric field, can only be performed using several approximations. The drift-diffusion process consists of two components: the initial stage of carrier extraction through carrier drift and the later current flow stage, sustained by diffusion supplied excess carriers from the electrically neutral bulk.

The initial stage of a current pulse is described by scalar representation of the acting electric field within a half of inter-electrode gap (0 ≤ *x* ≤ *d*/2) under a surface charge (σ/2) (sketched by small circles in Figure 3) induced by a fixed external voltage (*U*/2) and by excess electron concentration *n_0_* as:

(1) $$E(x)=-\frac{1}{\varepsilon \varepsilon_{0}}(\frac{\sigma }{2}+en_{0}x)$$

Here, symmetry, based on charge conservation at electrodes, is assumed, and this symmetry enables to consider a half of a system. Therefore, halves of σ, *U* and *d* are taken into account. By taking the second Poisson integral:

(1A) $$-\frac{U}{2}=\int_{d/2}^{d/2-X_{e}}E(x)dx$$

the surface charge σ is related to *U*, *n*_0_ and to an instantaneous position *X_e_* for the extraction of electrons, which density *n*_0_ is equal to that *p*_0_ of the homogeneously in-depth injected holes, as:

(2) $$\sigma =en_{0}d-\frac{\varepsilon \varepsilon_{0}}{X_{e}}U-en_{0}X_{e}$$

Extraction of electrons persists till then the charge σ on electrode is screened (an evolution of energy diagrams under a sequence of excitation pulses in a capacitor sensor biased by a relatively low voltage is sketched in Figure 4 where injected excess carriers are shown by large circles). This condition σ *=* 0 determines a quadratic equation for evaluation of the depletion depth *x* = *X_e0_*. A negative root of Equation (2) with σ *=* 0 leads to:

(3) Xe0(n0,U)=d2(1−1−εε0Uen0(d2)2)≈d2(εε0(U/2)en0(d2)2)

The last approximation in Equation (3) is valid if *X_e_*_0_ << *d*/2, at rather low voltages. In Equation (2), a pole for *X_e_* ≡ 0 appears if a diffusive outspread is ignored. This pole can also be circumvented by assuming a δ-thick skin-layer on metallic electrode (with initial coordinate *X_e_* − δ *=* 0 and δ*→*0, as in depletion approximation [41]). Actually, an external circuit always limits the rate of the current rise. Therefore, the infinities never appear in experimental situations. Equation (3) implies that the approximation of the bulk injected domain is relevant when an external field *U/d << en*_0_*(d*/2*)/*εε_0_ is weak relative to that created by a surface charge at a cross-sectional planes (at boundaries of the electrically neutral region (ENR) centred at *d*/2, Figure 4). This also means that the inequalities δ *< X_e_*_0_ *< d*/2 are held. 

The current variation in time during the initial stage of pulse (*i*_1_(*t*)) is then obtained (by differentiating in time the surface charge σ integrated over an area *S* of the electrode) as:

(4) $$i_{1}(t)=S\frac{d\sigma }{dt}=\frac{S}{X_{e}(t)}\left(\frac{\varepsilon \varepsilon_{0}U}{X_{e}(t)}-en_{0}X_{e}(t)\right)\frac{dX_{e}(t)}{dt}$$

Thus, to obtain the direct expression for the *i*_1_(*t*) function, the time dependent variations of the instantaneous positions (*X_e_*(*t*)) of the extracted charge of surface density *en*_0_*X_e_* and the drift velocity *dX_e_*(*t*)*/dt* should be described. These dependencies are obtained by solving the kinetic equation for drift of carriers (for instance, of electrons):

(5) $$\frac{dX_{e}}{dt}=-\mu_{e}E(n_{0},U,X_{e})=-\mu_{e}\frac{1}{2\varepsilon \varepsilon_{0}}\left(\frac{\varepsilon \varepsilon_{0}}{X_{e}}U-en_{0}(d+X_{e})\right)$$

The last expression for acting electric field is obtained by inserting σ calibrated to *U* (Equation (2)) into Equation (1). As a current is determined by the changes in time of charge on electrode, the dimensionless instantaneous position ψ *= X_e_/d* is essential. Thus, Equation (5) is rearranged as:

(6) $$\frac{d\psi }{dt}=\frac{1}{2}\left(\frac{1}{\tau_{Mn0}}(1+\psi )-\frac{1}{\tau_{TOF}}\frac{1}{\psi }\right)$$

By integrating Equation (6), the re-arranged expression of function ψ(*t*) is obtained for the range of 0 ≤ ψ’ ≤ ψ and 0 ≤ *t*’ ≤ *t*. Here, the characteristic time of dielectric relaxation is denoted as τ*_Mn0_ = ε*_0_*ε/en*_0_μ*_e_*, and a dimensionless depth of depletion is ψ*_e_*_0_ *= X_e_*_0_*/d*. The expression of the transit time τ*_tr_* is obtained by integrating Equation (6) for the range of 0 ≤ ψ’ ≤ ψ*_e_*_0_ and 0 ≤ *t*’ ≤ τ*_tr_.* Both integrals are obtained of the type:

(7) $$\int_{0}^{\psi ,\;\psi_{e0}}\frac{\psi \prime d\psi \prime }{\psi \prime^{2}+\psi \prime -\frac{\tau_{Mn0}}{\tau_{TOF}}}=\frac{1}{2\tau_{Mn0}}\int_{0}^{t,\;\tau_{tr}}dt\prime$$

respectively. The solutions of Equation (7) can be expressed through the roots of the denominator function as:

(8) $$\psi_{\pm }=\frac{-1\pm \sqrt{1+4\frac{\tau_{Mn0}}{\tau_{TOF}}}}{2}$$

which lead to the transcendental Equation:

(9) $$\psi (t)=\psi_{+}\left[1-{\left(\frac{\psi (t)+\psi_{-}}{\psi_{-}}\right)}^{-\frac{\psi_{-}}{\psi_{+}}}\exp\left(\frac{t}{2\tau_{Mn0}}(\frac{\psi_{-}}{\psi_{+}}+1)\right)\right]$$

Finally, by inserting Equation (6) with solutions of the Equation (9) into Equation (4), the initial stage of current pulse is expressed for the time interval 0 ≤ *t ≤* τ*_tr_* as:

(10) $$i_{1}(t)=\frac{S\varepsilon \varepsilon_{0}}{\psi (t)d}U\frac{1}{2\tau_{Mn0}}\left[\frac{1}{\psi (t)}-\frac{\tau_{TOF}}{\tau_{Mn0}}\psi (t)\right]\times \left[1+\psi (t)-\frac{\tau_{Mn0}}{\tau_{TOF}}\frac{1}{\psi (t)}\right]$$

An identical solution should be obtained by analysis of the hole extraction at the opposite electrode due to correlated, bipolar drift of electrons and holes (Figure 3, Figure 4 and Figure 5). The correlated drift (τ*_tr,e_* ≡ τ*_tr,h_* = τ*_b_*) determines the charge conservation in the system. Possible inequality of carrier mobilities is self-adjusted by different depletion lengths *X_e0_* ≠ *X_h0_* and a shift of a peak within the depth distribution profile of excess carrier concentration (like in the case of ambipolar diffusion with different velocities of surface recombination [42,43,44], a peak is shifted towards a surface of the slower surface recombination). Thereby, the induced current (on the opposite electrode), due to extraction and drift of holes, can be considered as a displacement current (not an additional current) which completes a circuit. A capacitor type detector with an injected bulk domain of excess carriers really acts as a junction type, partially depleted detector. There the merged electrically neutral regions and a spatially separated junction contact appear (where electrodes play the role of the junction which separates excess carriers). It would be worth to note that recombination of excess carriers within regions nearby the electrodes (of width *X_e0_*, *X_h0_*) is suppressed by lack of the recombination counter-partners—as one type of the excess carriers is extracted to electrode by external electric field. 

Further evolution of the induced current (by including even the transitional layer between the depleted and neutral regions) can be iterated using methodology presented by Equations (1)–(10). However, this leads to the extremely cumbersome and complicated transcendental equations. Therefore, to simplify analytical consideration for the case of large density of excess carrier pairs shown by large circles in Figure 4c and Figure 6, the scaling (τ*_tr_ <<* τ*_D_*) of the characteristic times of drift τ*_tr_* and of ambipolar (with a coefficient *D_a_*) diffusion τ*_D_* ≌ *d_eff_^2^/*4π^2^*D_a_* (within an effective thickness *d_eff_ = d − X_e0_ − X_h0_*) can be employed (Figure 6). There, the initial, short stage of the current pulse evolution is described by *i*_1_(*t*) (Equation (10)). The slow further stage (Figure 6) of the current pulse evolution is governed by τ*_D_* during which the excess carriers are supplied by diffusion to the depleted layer *X_e0_*. There, an assumption of the infinite surface recombination velocity is acceptable, as the fast drift (τ*_tr_ <<* τ*_D_*) keeps *n*_0_ ≈ 0 in the depleted near-electrode region (as a boundary condition *n*_0_ = 0 for *d_eff_*, equivalent to an infinite surface recombination). Using this assumption, the diffusion governed component *i*_2_(*t*) of the induced current can be estimated by time varied surface charge on electrode due to varied in time excess carrier concentration *n*_0_(*t*) in Equation (2) as:

(11) $$i_{2}(t-\tau_{tr})=S\frac{d\sigma }{dt}≅S\frac{\partial [en_{0}(t-\tau_{tr})(d-X_{e0}-X_{h0})-\frac{\varepsilon \varepsilon_{0}}{X_{e0}}U]}{\partial t}=eSd_{eff}\frac{\partial n_{0}(t-\tau_{tr})}{\partial t}$$

Solutions for the time varied concentration of excess carriers, averaged over *d_eff_*, are well–known (e.g., [45]) from theory of parabolic equations (with first kind boundary conditions, *n*_0_*|_x = 0; x = deff_* = 0). These solutions for a single-dimensional approach are expressed as:

(12) $$<n_{0}(t-\tau_{tr})>{|}_{d_{eff}}=n_{0}(t=0)\sum_{k=0}^{\infty }\frac{8}{\pi^{2}}{\left(-1\right)}^{k}\frac{\exp\left(-\frac{4D_{a}\pi^{2}}{d_{eff}^{2}}{\left(2k+1\right)}^{2}(t-\tau_{tr})\right)}{{\left(2k+1\right)}^{2}}$$

As carriers are supplied to *X_e0_* by diffusion, the sign within the exponential term of Equation (12) should be replaced by the opposite one, *i.e*., concentration supplied by diffusion during partial diffusion times τ*_D,k_ = d_eff_^2^*/4*D_a_*π^2^(2*k* + 1)^2^ as *X_e0_* changes with time. By differentiating Equation (12), the current component *i*_2_(*t −* τ*_tr_*) with *t_2_* = *t −* τ*_tr_* is then re-written as:

(13) $$i_{2}(t_{2})=eSd_{eff}n_{0}(t=0)\sum_{k=0}^{\infty }\left(\frac{8}{\pi^{2}}{\left(-1\right)}^{k}\frac{\exp\left(\frac{4D_{a}\pi^{2}}{d_{eff}^{2}}{\left(2k+1\right)}^{2}t_{2}\right)}{{\left(2k+1\right)}^{2}}\frac{1}{\tau_{D,k}}\right)$$

Thereby the entire current evolution within a transient is described (using the mentioned scaling) by Equations (10) and (13) as:

(14) $$i(t)=\{\begin{array}{c} i_{1}(t){|}_{0\leq t\leq \tau_{tr}}\\ i_{2}(t-\tau_{tr}){|}_{t>\tau_{tr}} \end{array}$$

Taking the carrier recombination within electrically neutral range (with recombination lifetime τ*_R_*) into account, the additional current components should be introduced as:

(15) $$i{\left(t\right)}_{+\tau_{R}}=\{\begin{array}{c} i_{1}(t){|}_{0\leq t\leq \tau_{tr}}-eS\frac{\partial (n_{0}e^{-\frac{t}{\tau_{R}}})}{\partial t}X_{e}(t)\\ i_{2}(t-\tau_{tr}){|}_{t_{2}>\tau_{tr}}+eSd_{eff}n_{0}(t=0)e^{-\frac{t_{2}}{\tau_{R}}}\sum_{k=0}^{\infty }\left(\frac{8}{\pi^{2}}{\left(-1\right)}^{k}\frac{\exp\left(\frac{4D_{a}\pi^{2}}{d_{eff}^{2}}{\left(2k+1\right)}^{2}t_{2}\right)}{{\left(2k+1\right)}^{2}}[\frac{1}{\tau_{D,k}}-\frac{1}{\tau_{R}}]\right) \end{array}$$

The simplified approach exploited describes well the qualitative experimental observations [46]: (i) at large densities of injected carriers (even for the localized initial domain) profiling of current transients by varying applied voltage leads to prevailing amplitude of the second component *i*_2_ attributed to diffusion (as the externally introduced charge on electrodes is screened by the injected excess charge, Figure 6); (ii) at moderate densities of excess carriers within the injected bulk domain (Figure 4c and Figure 6) and at relatively low applied voltages both components are observable, while the initial current *i*_1_ component prevails due to 1*/*ψ(*t*) for ψ(*t*) *<<* ψ*_e0_*(*t*) (Equation (10)); (iii) both components can also be observable (Figure 5 and Figure 6) for the perpendicular injection regime (for the excitation laser beam relative to field direction [47]) if an outspreading excitation beam overwhelms the inter-electrode gap; (iv) in voltage dependent profiling of transients both components overlap when threshold value of the applied voltage is sufficient to separate the electron and hole sub-domains over the entire inter-electrode gap.

### 3.2. Models for Injection of a Localized Domain

For the injection of a localized electron (*q_e_*)-hole (*q_h_*) domain, the relation between the surface charge (+σ*)* on the high potential electrode and the external voltage *U* is obtained by taking the second Poisson integral. The solution for a scalar surface charge σ depends on the instantaneous positions (ψ*_e_*(*t*) and ψ*_h_*(*t*)) of the drifting e-h sub-domains (Figure 5) within an inter-electrode gap of length *d.* The detailed consideration of the currents induced by the injected carrier capture and drift in capacitor and junction type sensors is presented in [38,39,40,48]. There it had been shown that induced instantaneous current values depend on the injected charge amount and on transit time which is consequently determined by the characteristic times of dielectric relaxation τ*_Mq e,h,_ =* ε*_0_*ε*d/q_e,h_*μ*_e,h_* and of free flight τ*_TOF,e,h_ = d^2^/U*μ*_e,h_* of the sub-domains of injected carriers with mobilities μ*_e,h_* (where carrier scattering is included within μ*_e,h_* and *D_a_* parameters)*.* Thereby an instantaneous velocity and the transit time depend on the amount of injected charge (*q_e,h_*). Also, current depends on time variations of the instantaneous positions of sub-domains (ψ*_e,h_*(*t*))*.* The latter dependence ψ*_e,h_*(*t*) is significant when the injected charge can be captured by defects. Then, the expression for current variations in time for the included carrier capture processes is represented as:

(16) $$i(t)=\frac{d\sigma }{dt}S=[-\frac{\partial q_{e}(t)}{\partial t}(1-\psi_{e}(t))-q_{h}(t)\frac{d\psi_{h}(t)}{dt}-\frac{\partial q_{h}(t)}{\partial t}\psi_{h}(t)-q_{h}(t)\frac{\partial \psi_{h}(t)}{\partial t}]S$$

for the mixed regime containing stages of the bipolar and monopolar drift, the initial velocity of holes (electrons) should be evaluated and the re-calibration of charge on the electrodes should be performed as described in [38,39,40], to satisfy the conservation of charge and charge momentum (*qv*). This gives a coincidence of *v_0,h,mon_* and *v_∑bip_*|_ψh_^*0^ values at position ψ_h_^*0^ of the hole domain. The generalized expression for current, attributed to the bipolar drift prolonged by the monopolar drift of holes, is represented as:

(17) $$i(t)=\{\begin{array}{c} i_{1}=\frac{qS}{\tau_{tr,e}}[\psi_{0}\exp(-\frac{t}{\tau_{tr,e}})+(1-\psi_{0})],\text{ for }0\leq t\leq \tau_{tr,e}=\tau_{bC};\;\\ i_{2}=\frac{q_{h}S}{\tau_{Mq,h}}\exp(\frac{t}{\tau_{Mq,h}})[\frac{v_{0,\Sigma bip}}{d}\tau_{Mq,h}+\frac{\tau_{Mq,h}}{\tau_{TOF,h}}-1],\text{ for }0\leq t\leq \tau_{tr,h}\equiv \tau_{tr,h,mon} \end{array}\;$$

The duration of the entire pulse (*t_P_*) is obtained as a sum of the bipolar drift τ*_bC_* and the hole domain drift τ*_dr,h,mon_*, as *t_P_ =* τ*_bC_ +* τ*_dr,h,mon_*. Similar solutions are obtained for electron prolonged drift for the mixed bipolar-proceeded by electron monopolar drift process. In the case of small charge drift, only an approximate analytical description of the process is possible, and the more rigorous consideration can be performed by including the retardation and magnetic field effects [49,50,51,52,53,54].

In the case of pure bipolar drift, a drift velocity appears to be invariable due to ψ*_e,h_*(*t*)*~t.* This leads to a square-wave shape of the current pulse. The real evolution (the rise to peak) of the current should be considered by including the external circuit parameters for the CUD (*C_d_*) capacitance charging process. The current decreases after the initial peak for the mixed drift regime, due to drag of a late arrived sub-domain by the counter-partner sub-domain. The phase of the monopolar drift only contains the increasing (if τ*_TOF,h_ =* τ*_Mq,h_*) or nearly constant (if τ*_TOF,h_ <* τ*_Mq,h_*) component of velocity. Thereby the double peak current pulse can be inherent for the mixed (a bipolar changed by a monopolar) drift regime. These drift prevailing regimes of current pulse formation are inherent for injection of sharply localized domains and even for bulk domains overwhelming the entire inter-electrode gap at sufficiently large voltages, capable of separating the initially neutral domain into the electron-hole sub-domains. In the latter case, the model of drifting (of localized plane—lateral surface charge plane) charge sub-domains can be acceptable, if the time of carrier grouping (increase of local carrier concentration) is shorter than the transit time of the drifting sub-domain.

## 4. Experimental Results and Discussion

### 4.1. Effects of the Excess Carrier Photo-Generation, Recombination and Surface Charging

In these experiments excitation quanta of less energy (*E_ph_* = 3.50 eV and 2.33 eV) than the forbidden energy-gap in diamond (*E_G_* = 5.46 eV) were used. Thereby, the excess carrier domains can be generated either by non-linear absorption or via photo-ionization of filled deep traps. Therefore, for separation of prevailing mechanisms of photo-excitation, it is important to clarify whether the monopolar or bipolar (as carrier pair) initial generation is implemented. The non-linear absorption processes of two-photon via virtual states or two-step absorption through empty deep levels are most probable processes. It had been proved [35] that both two-photon and two-step processes compete in generation of excess carrier pairs in defect-rich HPHT diamond samples at elevated excitation intensities. It was revealed that photo-ionization of filled deep traps can be performed by 2.33 eV quanta in defect-rich HPHT diamond samples [35]. This leads to a nearly linear dependence of the optical transmission signal on excitation intensity. The carrier pair generation has been implied from these measurements where electrons are generated from donor type deep traps while holes appear in valence band through rapid capture of electrons from valence band to the photo-emptied traps of large density. 

Optical transmission of the *d* thickness wafer sample can be described by a function:

(18) $$\frac{I_{0}}{I_{tr}}=\left(\frac{\text{α}+[\text{β}+{\text{β}}_{s}]P_{ex}}{\alpha }\right)\times \exp(\alpha d)\times \left(1-\frac{[\beta +\beta_{s}]P_{ex}}{\alpha +[\beta +\beta_{s}]P_{ex}}\times \exp(-\alpha d)\right)$$

of the excitation power density *P_ex_* and of the coefficients of the linear absorption α, of the two-photon absorption β and of the two-step absorption β*_S_* if all the mentioned photo-excitation processes appear simultaneously. In spectral range of sample transparency (for α*d* << 1 and α*d* < (β+ β*_S_*)*P_ex_d*), this expression can be simplified as:

(19) $$\frac{I_{0}}{I_{tr}}-1\approx (\text{β}+{\text{β}}_{S})P_{ex}d$$

Then carrier excitation rate is related to the absorption and laser pulse parameters as:

(20) $$n_{0}\approx [\alpha +(\beta +\beta_{S})P_{ex}]I_{0}$$

**Figure 7:**
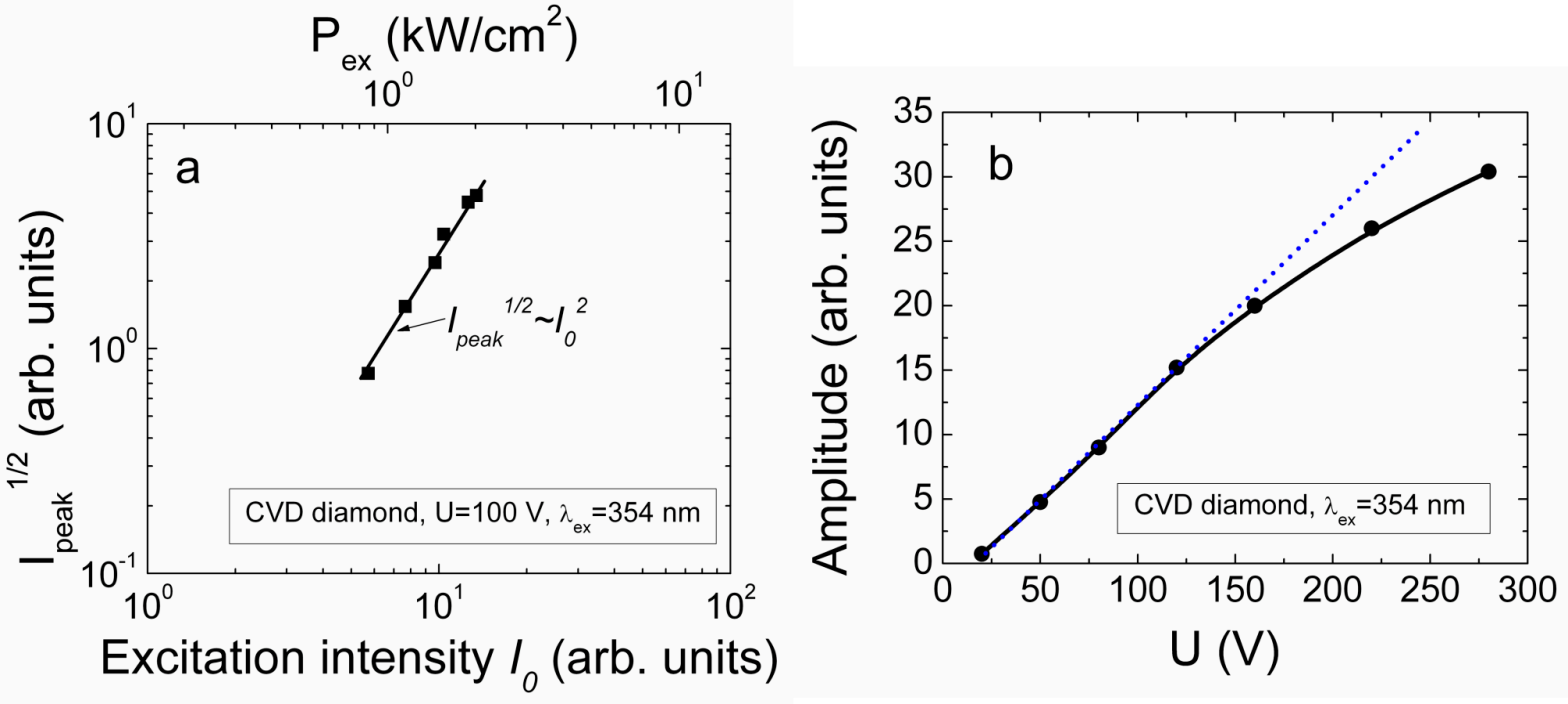
(**a**) The current peak amplitude as a function of excitation intensity at fixed value of applied voltage. The top scale indicates the range of applied power densities; (**b**) The current peak amplitude as a function of applied voltage for the fixed excitation intensity. Both characteristics were measured for CVD diamond by using injection of bulk domain with UV excitation wavelength λ*_ex_* = 354 nm.

The concentration generated at the end of a laser pulse τ*_L_* (in the case of fast recombination τ*_L_* ≤ τ*_R_*) is evaluated by the convolution integral averaged over laser pulse duration τ*_L_* expressed as:

(21) $$n_{0}(t=t\prime -\tau_{L}=0)\approx \frac{1}{\tau_{L}}\int_{0}^{t\prime }n_{0}\exp(-\frac{{\left(\Theta -\tau_{L}\right)}^{2}}{b\tau_{L}^{2}})\exp(-\frac{t\prime -\Theta }{\tau_{R}})d\Theta$$

here, *b* is a dimensionless parameter characterizing a temporal width of Gaussian laser pulse. It has been obtained that *n*_0_(*t =* 0) varies as a function of excitation intensity *I_0_*: for HPHT samples, as *n*_0_(*t = 0)~I_0_* for the range of a threshold *I_0_* values sufficient to detect current transients. While it becomes *n_0_(t =* 0)*~I*_0_^2^ for HPHT sensors at elevated excitation intensities. For the CVD diamond samples, the *n*_0_(*t =* 0)*~I*_0_^2^ characteristic prevails over the entire range where current transient signals can be detected. This implies a higher CVD crystal quality corroborated by the significantly longer carrier lifetimes in this material relative to the tested HPHT diamond samples. On the other hand, the concentration of the electrically active defects, attributed to the residual metals and nitrogen in the HPHT diamond material [35], exceeds that of CVD diamond [37] by more than four orders of magnitude. Additionally, owing to the longer carrier lifetime in CVD diamond, the peak amplitude of the initial component of the current pulse has been examined as a function of excitation intensity keeping a fixed value *U* of applied voltage to capacitor type detector (Figure 7). As can be deduced from Equations (3) and (10), the current peak value *i*_1,*peak*_ is proportional to ~(*Sεε_0_U*/2*d*ψ*)*τ*_Mn0_*^−1^ ≈ (*Sεε_0_U*/2*X_e0_*)τ*_Mn0_*^−1^* =* (*e*μ*_e_SU/X_e0_*)*n*_0_ and *X_e_*_0_*~n*_0_^−1^, *i.e*., *i*_1,*peak*_*~n*_0_^2^.

The measured dependence of *i*_1,*peak*_ on excitation intensity (for CVD diamond detector of capacitor type with injected bulk domain) is presented in Figure 7a. This dependence corroborates *n*_0_(*t =* 0)*~I*_0_^2^, *i.e*., the excess carrier generation through the two-photon absorption dominates in CVD diamond. The range of the excitation power densities *P_ex_* (upper scale on Figure 7a) from the side of low values is there limited by sensitivity of the measurement system to record current signals. Due to steep increase of the current peak values (*I_peak_~P_ex_*^4^*~I*_0_^4^) and necessity to keep the linearity of electrode characteristics, the dynamic range for *P_ex_* is also limited from the top side. Additionally, the upper range for values of the excitation power densities *P_ex_* can be shifted by using laser beam focusing. However, surface damage of diamond wafers can easily be reached (especially for HPHT samples). 

The signal dependence on applied voltage for the fixed excitation intensity appears to be a linear function (Figure 7b) over a wide range of voltages. This can be explained by a relation *i*_1*,peak*_*~*(*Sεε_0_U*/2*X_e0_)*τ*_Mn0_*^−1^*~U*. However, the change of the current enhancement slope with applied voltage within *i*_1*,peak*_*~f(U*) characteristic can be observed in Figure 7b, at the highest voltages exploited. This effect of *i*_1*,peak*_*~f(U*) slope change (similar to a current saturation effect) can be explained by competition of voltage dependent changes of *i*_1,*peak*_*~U*/*X_e0_* (Equations (4) and (5)) and of *X_e0_~U* (Equation (3)). This effect is also similar to a change of an I-V characteristic going from partial depletion regime to that of above full depletion voltage regime, within junction structures. Thereby, the saturation effect is determined by the significant separation of the injected sub-domains by the external voltage caused electric field.

**Figure 8:**
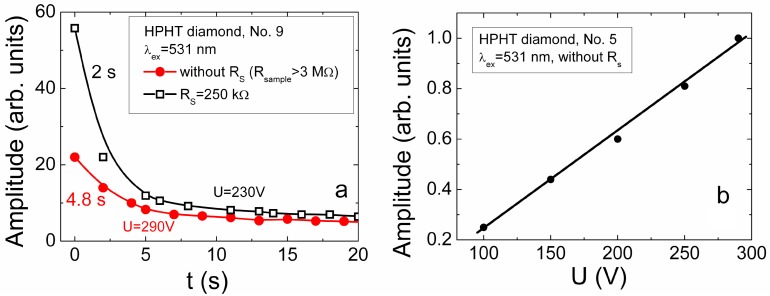
(**a**) Evolution of the current peak amplitude just after sharp switch-on a voltage of 290 V without (solid symbols) and with (hollow symbols) shunt resistance, measured in capacitor type detector made of HPHT diamond; (**b**) The current peak amplitude as a function of applied voltage for the fixed excitation intensity. These characteristics were measured by using injection of bulk domain at excitation wavelength λ*_ex_*= 531 nm.

**Figure 9:**
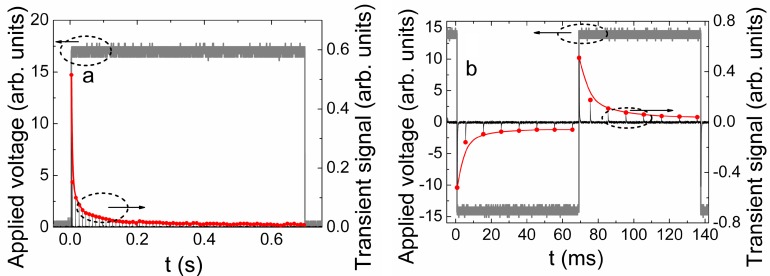
(**a**) A sequence of the charging current amplitudes (right scale) for a 700 ms pulse of the unipolar bias (16 V) voltage (left scale) applied to CVD diamond. The solid (red) curve is an eye-guide which shows the charging current decrease after each laser pulse of duration 0.4 ns in sequence of 70 laser pulses; (**b**) A sequence of the charging current amplitudes (right scale) for the 70 ms voltage pulses of the bipolar ± 14 V bias voltage (left scale) applied on CVD diamond capacitor. The solid (red) curve is again an eye-guide which shows the charging current (as vertical lines in this display scale) reduction after each laser pulse of duration 0.4 ns in sequence of 7 laser pulses (with 100 Hz repetition rate) for the negative and the positive polarity of bias voltage, respectively.

A polarization effect, similar to that reported in [13,16,28,29,30,31], has been unveiled in diamond samples. This effect (Figure 8a and Figure 9) appears as a decrease of the current peak amplitudes under action of a set of light pulses with invariable switched-on DC voltage. Actually the current signal nearly disappears using the same injection conditions if a capacitor type detector is kept with unipolar applied voltage of moderate values for a long exposure to a set of the injection light pulses with rather high repetition rate.

This polarization effect appeared to be independent of conditions of capacitor formation on diamond samples—the same phenomenon was inherent for both the mosaic electrode system containing sample No. 5 of the HPHT wafer set and the two-component metallization as well as the single copper metal, pressed electrodes on samples No. 7 and No. 9 [35] (Figure 1 and Figure 2). The polarization effect was observed for all the investigated HPHT and CVD diamond samples, being a little bit stronger for HPHT samples. This effect can be slightly suppressed by increasing of the applied voltage value. Therefore, evolution of the current peak amplitudes in profiling measurements has been examined just after a sharp voltage switch-on, *i.e*., ascribed to a single light pulse, to keep nearly the same acting electric field. The recovery of the acting electric field can be implemented either by a short-circuiting of the device electrodes or by applying the opposite polarity voltage. In the latter case, the capacitor charging current pulse of the opposite polarity is inevitably observed. Therefore, examination of the drift current transients is preferential at elevated applied voltages of moderate and large values, in order to reduce the role of the polarization effect.

The recorded evolution of the current peak amplitude in HPHT diamond structure just after a sharp switch-on of the moderate values of the applied voltage is illustrated in Figure 8a for 531 nm excitation wavelength. However, this effect was found for both 531 and 354 nm excitation wavelengths. The nearly linear dependence of the current peak amplitudes on the applied voltage have been obtained for the HPHT diamond detectors (Figure 8b). This characteristic proves the non-injecting electrodes over the range of applied voltages and of densities of the injected excess carriers. Therefore, the carrier trapping/recombination component has been hypothesized within induction currents in HPHT diamond samples. The linear excess carrier excitation regime can also be implied from these characteristics, Figure 8b. Then, formation of current transients can be described by the additional, carrier trapping component in Equation (17). In the reported approaches (e.g., [16]), the polarization effect is exceptionally interpreted assuming only this carrier trapping/de-trapping current component caused by non-movable space charge formation. Rapid trapping of the excess carriers onto deep centres can lead to the formation of the localized non-movable charge within *X_e0_* region. This localized charge in *X_e0_* keeps the induced charge Δσ at electrode till then the excess carriers are thermally/optically de-trapped from the deep centres to conduction/valence bands. The long de-trapping time from these deep centres can be a reason for a rather long relaxation (Figure 8a) of the quasi-stationary polarization field. Such characteristics and their interpretation are compatible with reported results and model in [16]. To verify whether this polarization appears due to bulk localized charge within *X_e0_* or via charge induction at electrode, the bypassing circuit was employed using a shunt resistance (Figure 2a) connected in parallel to the device under test. 

The shunt resistance was chosen to be large enough in order to keep nearly the same voltage drop on the inter-electrode gap, while the shunt resistance value was significantly less than that of the diamond sample. Verification of polarization was based on possibility to drain the induced charge Δσ from electrodes through shunt resistance, which is tunable to change the bulk localized charge within *X_e0_* till then the de-trapped carriers are brought from the *X_e0_* by their drift. The cumulated charge on electrodes can additionally be a reason for shielding of the external voltage source field. These measurements showed (Figure 8a, upper curve) that the current signal really increases and relaxation becomes faster, if shunt resistance is connected, relative to that without bypass circuit when other experimental conditions are kept the same. Thereby, it can be tentatively assumed that the polarization effect is caused by a cumulating of the induction charge on electrodes due to excess carrier localization at deep centres. Then, the space charge within widths *X_e0_*, *X_h0_* can appear due to charging of deep traps by captured excess carriers. This bulk localized charge determines the slow component of recharging through excess carrier de-trapping.

These observations prove a hypothesis of the induced charge Δσ on the electrodes and its accelerated extraction by partial shunting. These changes of relaxation rate also imply a dynamic component of the polarization effect attributed to free excess carriers (Figure 3c and Figure 4). A current transient, originated from a single light pulse through induction of surface charges at electrodes, may contain two components (Equation (16)) for each type of excess carriers: caused by the excess carrier drift and by the changes in time of the excess charge amount via carrier capturing into deep traps. As a result, the extracted charge at electrode appears (through fast drift component) which prevents further extraction of the same type carriers. Really, the external battery determines flow of currents by balancing the induction charges, to keep invariable external voltage. In consequence of surface and bulk charges, the internal electric fields appear, those are able to compensate the external source determined field. Thereby, the resulting electric field, acting on excess carriers, changes under cumulated excess carriers, if repetition rate of the injection light pulses is high in comparison with dielectric relaxation rate (Figure 8a). The polarization effect can alternatively be explained (Figure 4) by free carriers accumulated within regions of electrodes (polarity of which, for instance, positively charged contact for excess holes prevents extraction of free non-equilibrium holes to electrode) and separated by electrically neutral region at low applied voltages. This hypothesis seems to be supported by the existence of the polarization effect irrespective of the significant difference in concentration of deep traps in HPHT and CVD diamond, due to the fact the electrically active impurities (e.g., concentration of nitrogen (N) in HPHT diamond is of about 2 × 10^19^ cm^−3^ while it is less by a few orders of magnitude in CVD diamond) those would be the reason for the localized bulk charge. Recombination of accumulated excess carriers in the region between the electrically neutral region (ENR) and the electrodes is prevented due to a lack of the annihilation counter-partners (for instance, excess electrons are always extracted by external field at positively charged electrode while excess holes are accumulated). The capacitor charging current (determined by the external battery) can then be reduced (after each injection light pulse) by increase of *X_e0_* and *X_h0_* (Figure 4 and Figure 9), where internal voltage compensates the battery’s voltage (more and more after each pulse within a set of laser pulses). The sequenced generation of additional excess carriers with rather high rate of laser pulse repetition might then cause a cumulating of the induced charge Δσ at electrode. It also allows us to understand why the polarization effect is independent of surface metallization of the same set of samples, as the induced charge Δσ at electrode is not caused by ions collected on surface from atmosphere and from surface traps, as well. Thus, recovery of the applied field is only possible by reversing polarity of the external battery (when surface charges cumulated at electrodes are rapidly extracted from electrodes).

To clarify the dynamic component of polarisation field, experiments with pulsed external voltage source have been performed using a scheme shown in Figure 2c. There either a unipolar voltage pulse, of relevant duration (Figure 9a), or meandrous sequence of the two-polarity square-wave voltage pulses (Figure 9b) were exploited. The pulse duration was chosen to cover a set of identical laser excitation pulses running with 100 Hz repetition rate. Then the set of current transients flowing through load resistance from a pulsed voltage source was recorded. The first pulse in this sequence is attributed to the charging current of the capacitor under test by external voltage pulse. The external voltage pulse generator is triggered by an intermediate pulse generator (which is used for gating of the external voltage pulse duration, synchronized with a laser pulse driver). Thus, a set of current short pulses, ascribed to current transients originated from carrier injection laser pulses, is recorded. The sequence of the latter short pulses then appears as vertical lines within a time scale of external voltage source (Figure 9). The charging current decreases considerably under a sequence of excitation pulses, running with 100 Hz repetition rate, for unipolar *U_bias_* pulse, within similar to DC experiments (Figure 8a) relaxation time scale (Figure 9a). The accumulation process is only continued due to a sequence of injection laser pulses till an amount of accumulated charge reaches that value created by the external source. Therefore, every further injection pulse in the sequence determines the smaller charging current with approach of the accumulated charge carrier amount (under previous pulses) to that value of charge on voltage source electrodes, as observed in Figure 9a. The sudden change of voltage polarity leads to a rapid discharge (with sharp voltage decrease within rear phase of square-wave pulse) and fast charging of the discharged capacitor (Figure 9b). Then, the first pulse of the opposite polarity charging current (a photo-capacitance change phenomenon) reaches the same initial value (for a positive voltage pulse relative to the previous negative one). Afterwards, the decrease of charging current repeats, keeping symmetry of relaxation curves (Figure 9b). Thereby it can be inferred that polarization effect is determined by processes modelled in Section 3.1 (Figure 4), and it is originated from accumulation of the light injected excess carriers. Figure 9 also illustrates that relaxation rate is independent of the duration and polarity of bias voltage pulse while values of charging current decrease with applied pulsed voltage (when comparing Figure 9a,b). 

The polarisation effect is mostly manifested in the range of rather low voltages. However, it also depends on density of the injected excess carriers and injection (bulk or localized) regime. To have a recordable current signal at rather low voltages for bulk excitation regime covering the entire inter-electrode gap, a considerable amount of excess carrier pairs is needed, as the *i*_1,*peak*_ signal value decreases with reduction of voltage due to reduction of surface charge *n_0_X_e0_* (Figure 4). The nearly linear current *i*_1*,peak*_ dependence on applied voltage *U* (Equation (10), *i*_1*,peak*_*~*(*Sεε_0_U*/2*X_e0_)*τ*_Mn0_*^−1^*~U*) can be predicted in this range. The increased density *n_0_* of excess carrier pairs for a fixed low voltage may nevertheless enhance the dynamic polarisation effect (Figure 9). The latter leads to a reduction of *i*_1*,peak*_ through screening of the external field (which can be assumed to be equivalent to a reduction of the effective *U*) if the laser pulse repetition rate is rather high. The short current transient represents the excess carrier drift (if τ*_tr_* << τ*_R_*) or carrier recombination/trapping (if τ*_tr_* > τ*_R_*) dominated current components. For carrier recombination/trapping prevailing regime (more pronounced in HPHT diamond sensors), the current pulse duration is close to a recombination lifetime. For drift-determined current, the pulse duration should depend on applied voltage. Then, the polarisation effect of long relaxation component, determined by the space non-movable charge formation, is more probable. Therefore, the profiling of current transients in the range of low voltages was performed by measurements using a single injection pulse method, *i.e*., after acting electric field was recovered to the same value by manipulating polarity of applied voltage.

Enhancement of the applied voltage reduces the role of the polarisation effect in creation of the acting electric field (by weaker screening of the external source field). This leads to the increase of *i*_1*,peak*_*~*(*Sεε_0_U*/2*X_e0_)* with *U.* However, a width *X_e,h,0_* of depleted region simultaneously increases with *U*. The *i*_1*,peak*_ dependence on *U* starts to saturate if rates of the enhancement of *U* and of *X_e,h,0_*(*U*) become the same (Figure 8b). Further enhancement of *U* to values capable to separate excess carrier pairs, the components of carrier ambipolar (τ*_D_*) diffusion (which broadens the ENR region and supplies carriers to drift) and of drift (τ*_tr_*) can be observable (Figure 6), in case the carrier lifetime is properly long, (τ*_tr_ <* τ*_D_* << τ*_R_*). The latter components can be highlighted in the current transient profiling by applied voltage under bulk injection of excess carrier (either by parallel measurement geometry or by using the unfocused laser beams of diameter covering the inter-electrode gap in perpendicular measurement geometry, Figure 2a,b, respectively). 

For perpendicular geometry measurements (Figure 2b) with sharply focused laser beams, the profiling of current transients is therefore implemented by varying position of local injection at sufficiently elevated voltages, to highlight the carrier drift components. This voltage should thus be sufficient to separate electron and hole pairs. It was assumed in the above discussed situations that the density of injected carrier pairs is kept rather small, a little above the threshold value, necessary for the recordable currents. The enhancement of the injected carrier density leads, certainly, to an increase of the peak currents. However, the enhancement of excitation density was performed gently, especially for capacitor type sensors of a rather small area of the electrodes—the injected large density of carriers might be a reason for screening of external field (Figure 6). Then current contains pure convection component governed by ambipolar diffusion. The geometrical and the voltage as well as injection parameters should be properly chosen to perform correctly (by avoiding diffusion components) the profiling experiments, when using the perpendicular injection regime. 

### 4.2. Profiling of Current Transients in HPHT Diamond

Current transients have been profiled in HPHT diamond using two regimes: (i) by varying applied voltage when a fixed intensity excitation beam impinges through an electrode in parallel to the direction of electric field; and (ii) by varying location of a focused injection beam within the inter-electrode gap, where directions of electric field and of laser beam are perpendicular to each other.

**Figure 10:**
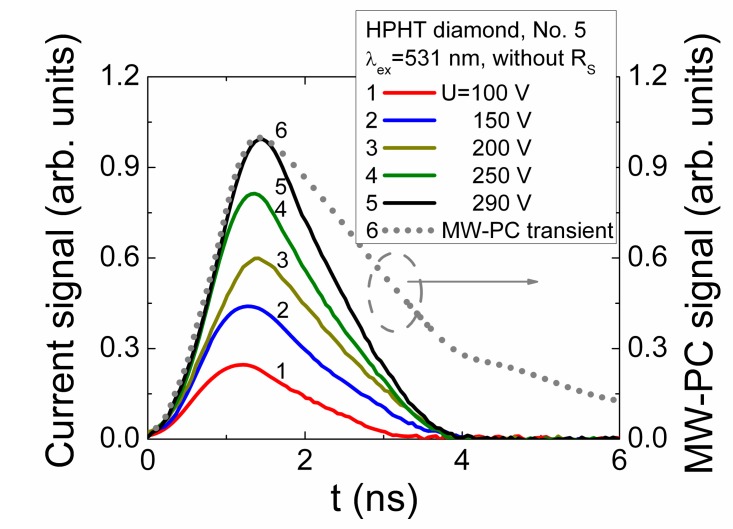
Variation of the induction current transients dependent on the applied voltage in a capacitor type detector made of a mosaic set of linear implanted contacts and metal electrodes containing HPHT diamond sample No. 5. Injection of excess carriers has been implemented by 531 nm light laser pulses impinging through hole in the electrode.

Variation of current transients obtained by changing the fixed values of applied voltage in capacitor type detector made of HPHT diamond sample No. 5 (Figure 1) is illustrated in Figure 10. Here, excitation by 531 nm light pulses was implemented. In this case, excess carrier generation from deep centres prevails and a bulk charge domain is injected. The shape of the induced current transients is inherent for the carrier recombination-drift dominated process (discussed in Section 3.2).

The initial current peak can be ascribed to the bipolar drift of the injected carriers, where pulse duration and further current decrease can be ascribed to carrier trapping/recombination. The excess carrier density relaxation transient, recorded without external electric field and measured by microwave probed photoconductivity (MW-PC) technique, is additionally shown in Figure 10 by dot curve. Here, the normalized amplitude of MW-PC signal is exploited to adequately scale the MW-PC and ICDC transients. The range (>100 V) of applied voltages (Figure 10) was sufficient to get a short (<2 ns) transit time for the drift-recombination of the injected carriers, where the role of the polarization effect is also reduced. The rather large applied voltage determines the sufficiently wide depletion regions (*X_e0_*, *X_h0_*) nearby the electrodes. Therefore the current component ascribed to extraction of the diffusion brought carriers is also rather short, and it overlaps with current component attributed to carrier drift. In order to separate different components of the induced current in time scale, where temporal resolution and duration of processes is of the same order of magnitude, the deconvolution of transients has been performed using the parameters of the external circuit and of excitation pulse.

However, it was not possible to clearly discriminate the components of a current transient due to short characteristic times (τ*_tr_*, τ*_R_*) which values are close to the temporal resolution of experimental system. This issue can also be implied from Figure 10, where no clear shift of the kink point relative to time axis, attributed to arrival of drifting carriers, is resolved when applied voltages have been varied significantly. Such a result hints the competition between the drift and recombination, assumed within equation (Equation (16)). The similar voltage dependent characteristics (transient shapes and temporal parameters) have been obtained for all the investigated HPHT diamond detectors under injection of bulk domains by UV light (354 nm) pulses. These results show that usage of the pressed plate electrodes (pinned to either bare polished wafer samples surfaces—No. 7 and No. 9, Figure 1—or to additionally metallized surfaces (sample No. 5, Figure 1)) exhibit the capacitor type detector features.

**Figure 11:**
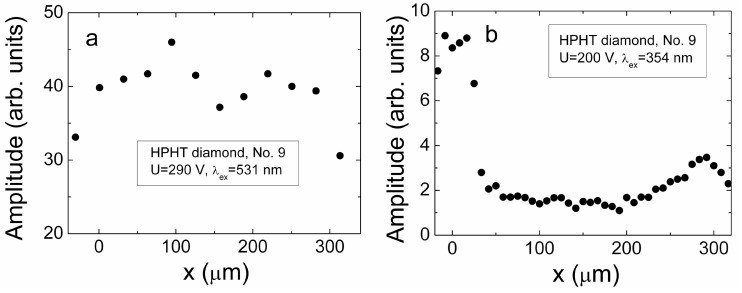
The cross-sectional profiles of the peak values of current induced by scanning location of a focused injection beam of 531 nm (**a**) and 354 nm (**b**) light, obtained for HPHT diamond sample No. 9.

The cross-sectional profiles of the peak values of current induced by scanning location of a focused injection beam of either 531 nm or 354 nm light, obtained for HPHT diamond sample No. 9, are illustrated in Figure 11a,b, respectively. This sample is rather homogeneous and contains a single growth sector of cubic orientation. A strip focusing regime implemented by a cylindrical lens enables to exclude the impact of the transverse carrier diffusion

**Figure 12:**
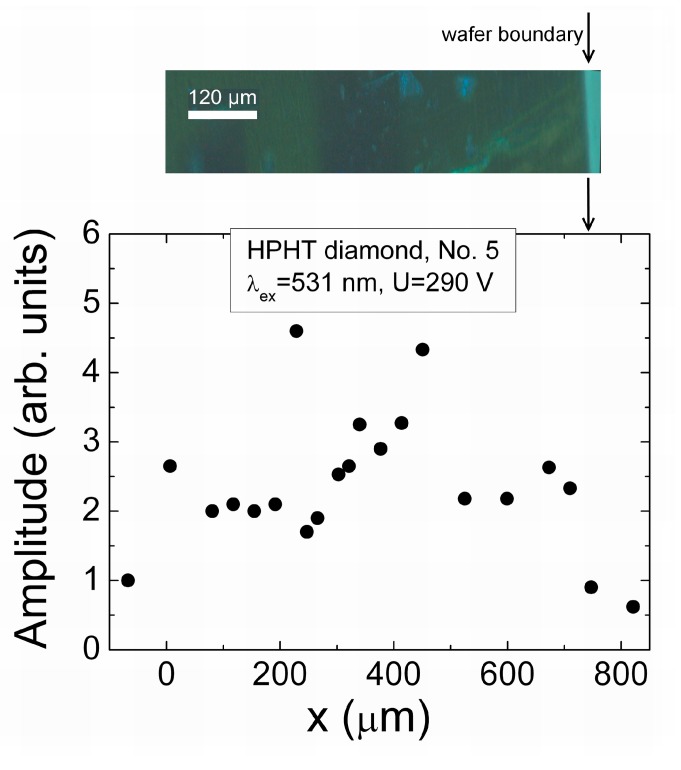
The cross-sectional profile of the peak values of current induced by scanning location of a focused injection beam of 531 nm light, obtained for HPHT diamond sample No. 5. The microscopy image of wafer boundary taken in UV light for the scanned area is shown on top of the current distribution profile plot, where the extended defects are imaged by luminescence spots (bright-blue) within bulk of wafer.

The profile scanned by using 531 nm (Figure 11a) light pulses contains a rather flat vertex. This indicates the recombination prevailed drift process. Only shallow sag within the profile vertex can be noticed which can be attributed to a current reduction due to the increased transit time. The profile unveiled by the two-photon bulk domain injection, when using 354 nm (Figure 11b) light pulses, has a clear relief. The peak values of the induced current are increased by approaching of injection beam location to electrodes. This indicates the prevailing of carrier drift. Then, the current increase at electrodes is explained by the shortest drift path and, thereby, the shortest transit time. Complementarily, these current values are the smallest ones for the injection beam locations in the mid of an inter-electrode gap. Carrier recombination also might be an additional reason for reduction of the collected charge when the drift time is close or even exceeds that of carrier recombination lifetime. The impact of carrier lifetime is the most prominent within cross-sectional profiles scanned on the depth-inhomogeneous HPHT diamond samples. Such an illustration is presented in Figure 12. This illustration (Figure 12) shows a cross-sectional profile of the induction current obtained for detector formed on the sample No. 5, where clear inclusion of metallic precipitates were visualized (by microscopy imaging—on top) within sample depth.

The cross-sectional profile (Figure 12) scanned for this HPHT diamond sample No. 5 shows a random distribution of the current peak values. There is neither enhancement of current due to reduction of transit time when injection location approaches to electrodes nor flat vertex ascribed to homogeneous distribution of defects, acting as the recombination centres. Such a profile qualitatively correlates with cross-sectional wafer image where defects of different size and origin can be resolved.

### 4.3. Profiling of Current Transients in CVD Diamond

**Figure 13:**
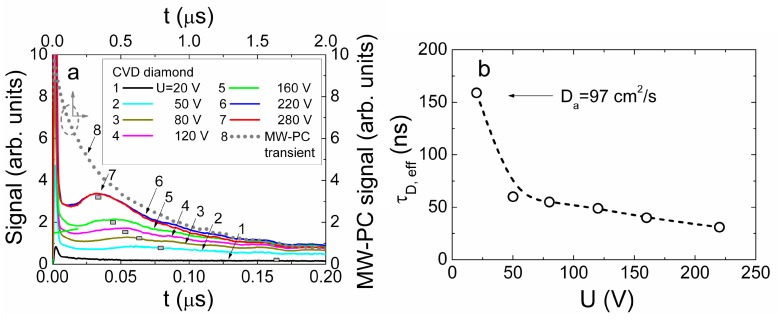
(**a**) Variation of the current transients recorded in the CVD diamond device by changing bias voltage when excess carrier bulk domain is injected through electrode using UV 354 nm light pulses. Dot-curve, ascribed to the top-right scales, represents relaxation of excess carrier density measured by microwave probed photo-conductivity (MW-PC) technique. It should be noted that the time scale (bottom) for the carrier drift-diffusion transients is considerably shorter than that (top) for MW-PC transient; (**b**) Diffusion ascribed effective time τ*_D_*_,*eff*_ (which is determined as a time shift between the current components (peak values *i*_1,*peak*_ and *i*_2,*peak*_) within a long scale of transient display) as a function of applied voltage.

The same (as for HPHT diamond) profiling regimes were applied for capacitor type detector made of CVD diamond. Owing to the considerably longer carrier recombination lifetimes (τ*_R_* ≥ 110 ns, Figure 13a) in CVD material, the main current components can be resolved by varying profiling regimes. Evolution of the induced current transients, recorded in CVD diamond device profiled at fixed excitation intensity by varying applied voltage, are illustrated in Figure 13a. The transients displayed within a sub-microsecond time scale enable observation of both current components, namely, *i*_1_ and *i*_2_, discussed in Section 3.1 (Equation (15)). The peak values of the drift current component *i*_1_ significantly exceed those attributed to the diffusion supplied carrier extraction component *i*_2_. Increasing of the applied voltage leads to an enhancement of a width of the depleted regions at electrodes and of the acting electric field, thereby reducing the time necessary to extract the diffusion supplied carriers. This determines a shift of the peak position ascribed to the *i*_2_ current component towards a time scale of the transit times with enhancement of applied voltage. Thereby, the parameter of an effective time τ*_D,eff_* ascribed diffusion can be introduced. This parameter is determined as a time shift between the current components *i*_1_ and *i*_2_ fixed by their peak values (*i*_1,*peak*_ and *i*_2,*peak*_). Variation of the drift-diffusion ascribed effective time τ*_D,eff_* as a function of applied voltage is illustrated in Figure 13b. It can be deduced from Figure 13b that this τ*_D_*_,*eff*_ time is weakly dependent on applied voltage for the range of elevated *U* > 50 V values, where the excess carrier drift prevails. Contrary, reduction of applied voltage below the *U* < 50 V leads to a sharp increase of τ*_D,eff_* up to 150 ns. Unfortunately, this time scale is in the range of the carrier recombination lifetimes in CVD diamond. Therefore, the *i*_2_ signal considerably falls down, and precision of separation of the *i*_2,*peak*_ is improper to proceed measurements at significantly lowered voltages. Nevertheless, τ*_D_* ≌ *d_eff_^2^/*4π^2^*D_a_* values, extracted for the voltage range *U* < 50 V, indicate a clear prevalence of diffusion process. This enables estimation of values of a coefficient *D_a_* of the carrier ambipolar diffusion in CVD diamond, assuming that current *i*_2,*peak*_ peak value is attributed to the main (regular regime [45]) mode (*k* = 0) of diffusion in sample with infinite surface recombination. The estimated value of *D_a_* = 97 cm^2^/s is in good agreement with those values calculated using the parameters of carrier drift mobilities, published in literature (e.g., [13]).

**Figure 14:**
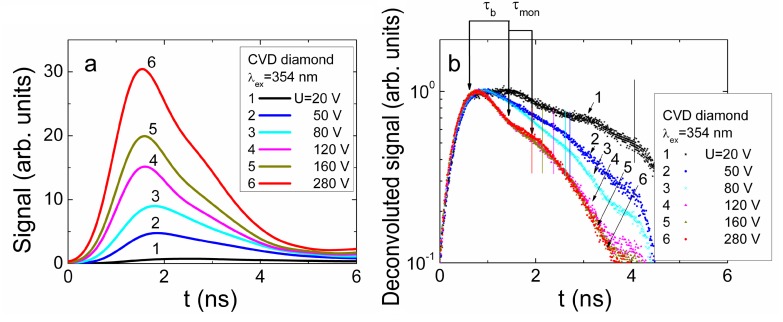
(**a**) Variation of the current transients recorded in short display scale by changing bias voltage when excess carrier bulk domain is injected through electrode using UV 354 nm light pulses; (**b**) The deconvoluted transients as a function of applied voltage. The bipolar (τ_b_) and monopolar (τ_mon_) drift components are denoted by arrows for drift of electrons within the final phase of transient at U = 280 V.

In order to examine the fast current component *i*_1_, current transients were recorded within short (a few ns) display scale, to enhance precision. Evolution of this transient component as a function of applied voltage is represented in Figure 14a. The asymmetric shape transients (different from those recorded for HPHT diamond), relative to a peak position, were obtained. Unfortunately, due to improper temporal resolution of the experimental arrangement exploited, direct analysis of drift characteristics is impossible. Therefore, the transients have been deconvoluted using Gaussian convolution function with parameters adjusted to the initial delay component correlated with the experimental transients. The deconvoluted transients are illustrated in Figure 14b. There the initial rise to peak becomes more abrupt. The rearward kink, ascribed to the arrival time of carriers to electrode, can be discriminated. This kink instant on vertex of a current pulse is employed to estimate the transit time which is measured between the initial peak (*i*_1,*peak*_, this indicates the end of excitation pulse), and the rearward instant of kink formation on vertex of current pulse (which indicates the end of drift). The instant (shown by vertical lines in Figure 14b) of a kink point depends on applied voltage, and it shifts towards the beginning of current pulse with enhancement of voltage. There the components of the bipolar and monopolar drift can tentatively be implied. However, the unambiguous attribution of these components to the paths of the hole and electron drift and to their transit times is very complicated in the parallel geometry (Figure 2a) measurement regime. Such changes again have a clear tendency only in the range of elevated voltages *U* > 50 V. For the range of *U* < 50 V, the amplitude of the deconvoluted transient (curve 1 Figure 14b) increases considerably together with its duration, and this implies an overlap of drift and diffusion governed components in the induced current transients which becomes improper for analysis of transit times.

**Figure 15:**
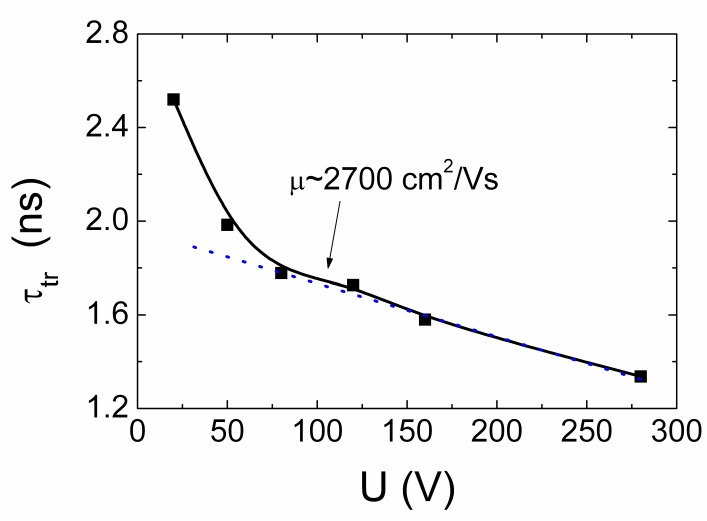
The transit time evaluated from the deconvoluted current transients as a function of applied voltage using an approximation of the flat vertex of pulse.

The measured effective transit time as a function of applied voltage is illustrated in Figure 15. The transit time τ*_tr_* increases almost linearly with reduction of applied voltage (Figure 15) for the range of voltages U = 75–300 V. There precision of evaluation of the τ*_tr_* falls down in the range of *U* > 200 V. There both the extraction depth and an amount of extracted charge become dependent on applied voltage and partially compensate variations of each other, as discussed in Section 3.1 and Section 4.1. Nevertheless, the roughly estimated value of carrier mobility μ *≈* [2*X_0_*^2^/(*U*τ*_tr_*)] *≈* 2700 cm^2^/Vs within correlated drift of excess carriers is in agreement with mobility values reported in literature (e.g., [13]). This evolution (Figure 15) of a short current component enables only estimation of the averaged carrier mobility at assumption of the flat vertex of current pulse from the measured transit time as a function of applied voltage. The assumption of the flat vertex of a current pulse is closely related to the steady-state models where charge supplied from voltage source during carrier drift (to simultaneously keep the invariable applied voltage and the charge conservation under varied CUD capacitance [38,39,40,48], due to additional light injected charge) is ignored. The widely exploited steady-state models are based on assumption of the independent (non-correlated) drift of holes as well as of electrons and on ignoring of the Shockley-Ramo’s effect—the induction of charge on electrodes due to moving externally injected carriers within inter-electrode space. There TCAD simulations can be exploited (for instance, [48]) for modelling of the carrier drift process in a steady-state approach. However, the simulated transients using the steady-state and the dynamic models [48] can considerably differ. In the steady-state approach, the histogram type drift current components for holes and electrons and of their sum appear, as usual. 

The assumption of non-correlated drift of holes and electrons, used in steady-state models, is equivalent to the increased drift path *d_∑_*, as *d_∑_ = d_e_ + d_h_ > d,* which exceeds the inter-electrode gap *d*. It can alternatively be misinterpreted that the double elementary charge *e*, *i.e*., *e* × 2, is carried during the bipolar drift of an electron-hole (e-h) pair. However, it can easily be verified (e.g., [48]) that an e-h pair carries (over the path of the bipolar drift) the same elementary charge *e* as either a single hole or electron, which proceeds the charge transport process after one counter-partner of a pair reaches the electrode. Thus, ignoring of the changes of the acting electric field, caused by charge supplied to electrodes from an external voltage source (if carrier injection from electrodes can be ignored for the junction and capacitor type devices), leads to the underestimated carrier mobility values, if *d_∑_ > d* is considered.

**Figure 16:**
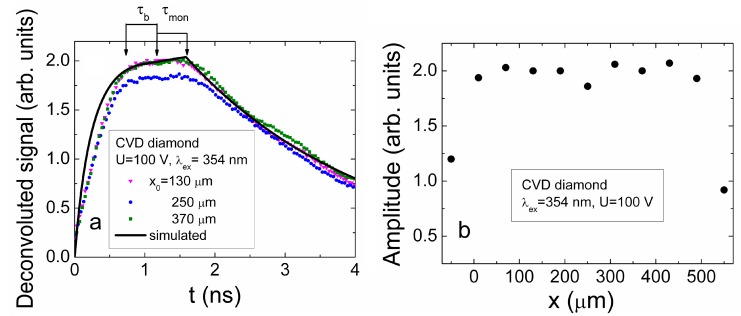
(**a**) The deconvoluted current transients for several injection positions recorded within cross-sectional scans of the CVD diamond capacitor type detector. The bipolar (τ_b_) and monopolar (τ_mon_) drift components are denoted by arrows for drift of electrons within the final phase of transient. The solid curve represents a simulated transient for injection location *x* = 130 μm relatively to the negative electrode at voltage U_bias DC_ = 100 V; (**b**) The amplitude of the induced current (ascribed to the bipolar drift component) as a function of the injection position within inter-electrode gap, measured in capacitor type detector made of CVD diamond.

In current transients recorded by the cross-sectional profiling of the charge injection location, the injection position and paths of electron and hole drift can definitely be correlated with electrode voltage polarity. In these current transients, recorded by cross-sectional profiling on CVD diamond sample, only a short current component has been observed ascribed to the drift of the separated electron-hole sub-domains. The deconvoluted transients are illustrated in Figure 16a. The components of the bipolar and monopolar transit times are there also denoted in Figure 16a. The simulated transients (as that illustrated in Figure 16a for a single transient) have been employed for evaluation of carrier mobilities using dynamic models mentioned in Section 3.2 and published in more detail in [38,39,40,48]. There parameters of the injected charge amount, of the external circuit, of carrier trapping/detrapping and other (ascribed to measurement geometry, voltage, *etc*.) have been incorporated. The fitting of the simulated transient to the experimental one at different applied voltages leaded to values of electron (μ*_e_*) and hole (μ*_h_*) mobility values of μ*_e_* = 4000 cm^2^/Vs and μ*_h_* = 3800 cm^2^/Vs, respectively. The close values of μ*_e_* and of μ*_h_*, nevertheless, lead to a rather flat vertex of the recorded transients. On the other hand, the non-ideality of the experimental conditions and temporal resolution of our experimental circuits complicated a perfect fit of the experimental and simulated transients. For instance, the initial and rear slopes within the deconvoluted transients indicate a non-abrupt strip boundary shape of the focused excitation beams. The separated values of μ*_e_* = 4000 cm^2^/Vs and μ*_h_* = 3800 cm^2^/Vs by using the cross-sectional profiling of the charge injection location exceed that value μ = 2700 cm^2^/Vs extracted from the voltage profiling on CVD diamond sample in parallel measurement geometry. The reason of these deviations, mentioned above, can be explained by non-adequate evaluation of drift paths when parallel measurement geometry is exploited.

The profile of the induced current amplitude as a function of this beam location also exhibits a rather flat vertex (Figure 16b). This profile can be understood at assumption of close values of μ*_e_* and of μ*_h_.* Nevertheless, a shallow sag within the profile vertex can there be noticed in Figure 16b which can be attributed to current reduction due to the increased transit time when excitation beam is localized in the mid of the inter-electrode gap.

## 5. Conclusions

Analytical models adequate to the experimental profiling regimes have been developed to interpret the evolution of current pulse transients induced by the bulk and localized domains of the injected charge carriers. The current components ascribed to the carrier drift, diffusion and recombination processes have been discriminated and interpreted. It has been demonstrated that the capacitor type detectors can be made of HPHT and CVD diamond by using pressed plate electrodes pinned to either the bare polished surfaces of wafer samples or to the additionally metallized surfaces, which exhibit the capacitor type detector features of fast responses.

The reliable discrimination of prevailing mechanisms in the formation of shapes and durations of the induced current pulses can be implemented by combining profiling of current transients by injection of the bulk and the localized excess carrier domains. It has been shown (for the examined HPHT diamond samples) that current transient profiling by the cross-sectional scans of the injection location can be a sensitive tool for identification of depth distribution of growth defects. The cross-sectional profiling of current transients enabled us to evaluate the impact of diamond crystal quality on the operational characteristics of the capacitor type detectors. However, short carrier lifetime in the examined HPHT diamond samples complicated discrimination and evaluation of carrier drift parameters. The combined profiling of current transients by injection of the bulk and of the localized domains of excess carriers enabled us to evaluate the carrier transport characteristics for CVD diamond. The electron (μ*_e_*) and hole (μ*_h_*) mobility values of μ*_e_* = 4000 cm^2^/Vs and μ*_h_* = 3800 cm^2^/Vs have been evaluated by fitting current transients recorded by the cross-sectional profiling of the charge injection location. The coefficient of carrier ambipolar diffusion of *D*_a_ = 97 cm^2^/s has additionally been determined in CVD diamond material from current transient voltage profiling. A mobility value of μ ≌ 2700 cm^2^/Vs for the non-correlated drift of the excess carriers was alternatively estimated from the voltage profiling of current transients using an approximation of the flat vertex of current pulse. Deviation of the latter μ value from those of μ*_e_* and μ*_h_* can be explained by non-adequate estimation of the carrier drift paths and the acting electric field. The near linear increase of current with applied voltage was obtained for transients examined in HPHT diamond samples. This can be explained by prevailing of the carrier capture component within transients of the injected charge currents. The seeming saturation of currents in the range of the largest applied voltages has been observed for the CVD diamond sample, that is routinely interpreted as a drift velocity saturation effect. However, this change of the current enhancement slope with applied voltage can alternatively be explained by the change of the carrier drift regime, which differs for partially and fully depleted (from excess carriers) inter-electrode gap.
